# High-temperature quantum oscillations of the Hall resistance in bulk Bi_2_Se_3_

**DOI:** 10.1038/s41598-017-18960-0

**Published:** 2018-01-11

**Authors:** Marco Busch, Olivio Chiatti, Sergio Pezzini, Steffen Wiedmann, Jaime Sánchez-Barriga, Oliver Rader, Lada V. Yashina, Saskia F. Fischer

**Affiliations:** 1 Novel Materials Group, Humboldt-Universität zu Berlin, Newtonstraße 15, 12489 Berlin, Germany; 2 High Field Magnet Laboratory, Radboud University Nijmegen, P.O. Box 9010, 6500 GL Nijmegen, Netherlands; 3 Helmholtz-Zentrum-Berlin für Materialien und Energie, Albert-Einstein-Straße 15, 12489 Berlin, Germany; 4 Department of Chemistry, Moscow State University, Leninskie Gory 1/3, 119991 Moscow, Russia

## Abstract

Helically spin-polarized Dirac fermions (HSDF) in protected topological surface states (TSS) are of high interest as a new state of quantum matter. In three-dimensional (3D) materials with TSS, electronic bulk states often mask the transport properties of HSDF. Recently, the high-field Hall resistance and low-field magnetoresistance indicate that the TSS may coexist with a layered two-dimensional electronic system (2DES). Here, we demonstrate quantum oscillations of the Hall resistance at temperatures up to 50 K in nominally undoped bulk Bi_2_Se_3_ with a high electron density *n* of about 2·10^19^ cm^−3^. From the angular and temperature dependence of the Hall resistance and the Shubnikov-de Haas oscillations we identify 3D and 2D contributions to transport. Angular resolved photoemission spectroscopy proves the existence of TSS. We present a model for Bi_2_Se_3_ and suggest that the coexistence of TSS and 2D layered transport stabilizes the quantum oscillations of the Hall resistance.

## Introduction

Among the new material class of topological insulators (TI), the chalcogenide semiconductor Bi_2_Se_3_ has been long subject to intense investigations due to its potential integration in room temperature applications, such as dissipationless electronics and spintronics devices^1–4^. Bi_2_Se_3_ has a single Dirac cone at the Γ-point in the first surface Brillouin zone and a direct band gap of 0.3 eV between the valence and the conduction band^5–7^. Due to the inversion symmetry in Bi_2_Se_3_ the topological *Z*_2_ invariant *ν* = (1;000) is equal to the charge of parity of the valence band eigenvalues at the time-reversal-invariant points of the first Brillouin zone caused by the band inversion^8^. In the crystalline modification Bi_2_Se_3_ has a tetradymite structure with R$\overline{3}$m symmetry. The unit cell consists of 15 atomic layers grouped in three quintuple layers with Se–Bi–Se–Bi–Se order stacked in an A–B–C–A–B–C manner. The quintuple layers are van der Waals bonded to each other by a double layer of Se atoms, the so-called van der Waals gap^4^. The existence of TSS in Bi_2_Se_3_ has been experimentally confirmed through angle resolved photoemission spectroscopy (ARPES)^3,7,9^ and scanning tunneling microscopy/scanning tunneling spectroscopy (STM/STS)^10,11^. The as-grown crystals of Bi_2_Se_3_ are typically *n*-type because of electron doping due to natural selenium vacancies^12,13^. Therefore, the transport properties of Bi_2_Se_3_ are generally dominated by bulk conduction. In particular, the temperature dependence of the electrical resistivity *ρ* is metallic-like^3,14–17^ and Shubnikov-de Haas (SdH) oscillations in the longitudinal resistivity *ρ*_xx_ show the characteristic signatures for a 3D Fermi surface^14,17^. For highly Sb-doped samples with lower carrier density $n\sim {10}^{16}$ cm^−3^, the TSS can be detected via additional SdH oscillations with a frequency *B*_SdH_ higher than that of the bulk and the Hall resistivity *ρ*_xy_ exhibits quantum oscillations for a carrier density *n* < 5 ⋅ 10^18^ cm^−3^ (ref.^18^). Different from that, for *n* ≥ 2 ⋅ 10^19^ cm^−3^ a bulk quantum Hall effect (QHE) with 2D-like transport behavior was reported^3,16^. Its origin remains unidentified.

In this work we demonstrate that the quantum oscillations of the Hall resistance *R*_xy_ in high-purity, nominally undoped Bi_2_Se_3_ single crystals with a carrier density of *n* ≈ 2 ⋅ 10^19^ cm^−3^ persists up to high temperatures. The quantum oscillations in *R*_xy_ scale with the sample thickness, strongly indicating 2D layered transport. These findings stand out because the Bi_2_Se_3_ samples investigated here have a lower carrier mobility *μ* of about 600 cm^2^/(Vs) than materials hosting a typical 2D Fermi gas^19–22^ or 3D Fermi gas^23–25^ showing QHE. We discuss the conditions of the QHE below in detail and present a model for the coexistence of 3D bulk, 2D layered and TSS transport.

## Results

### Experimental data

**Figure 1:**
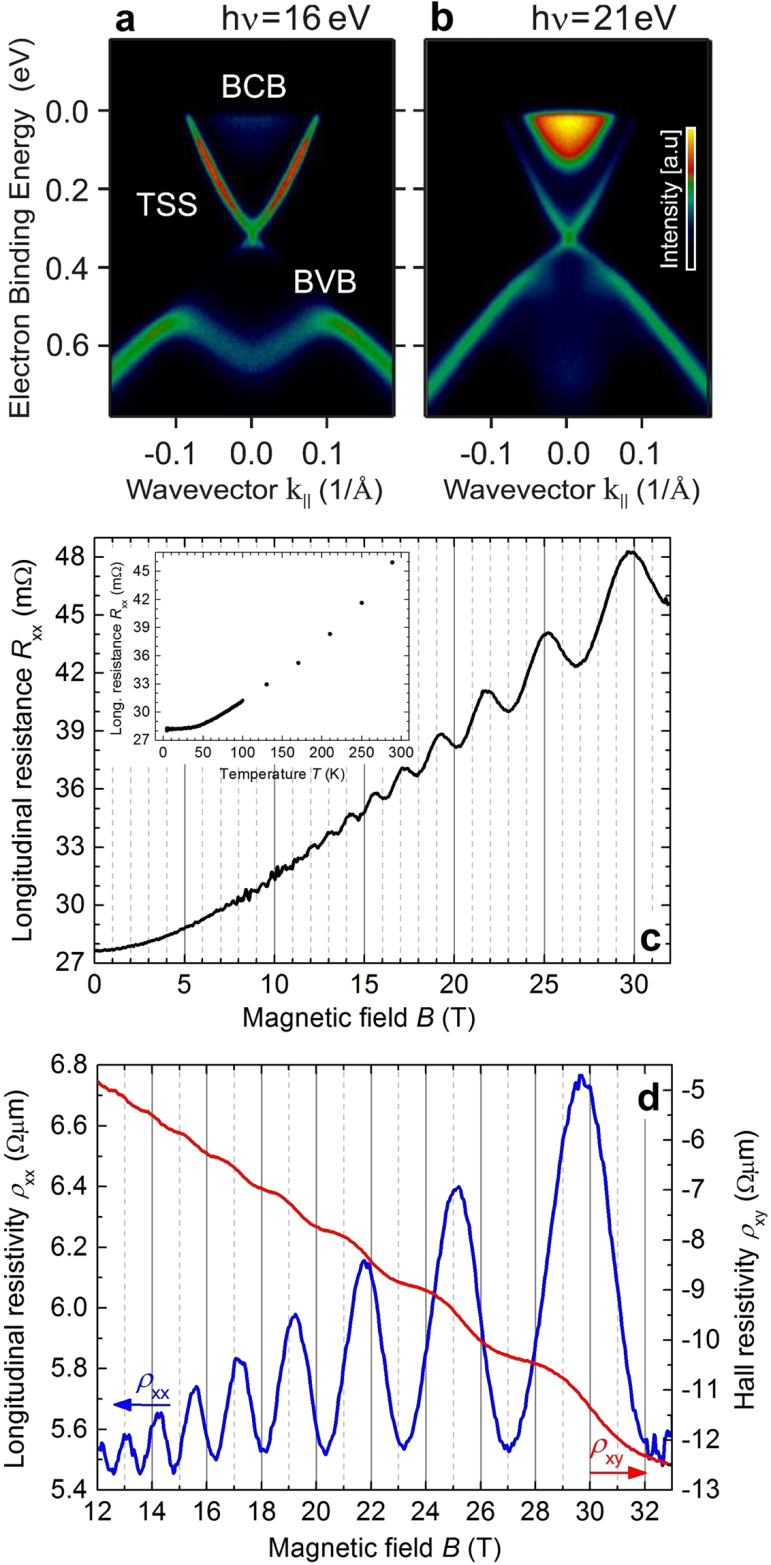
Electronic structure, temperature-dependent resistance and magnetotransport properties of Bi_2_Se_3_.(**a**) and (**b**) Electronic structure of the Bi_2_Se_3_ bulk single crystal before mechanical exfoliation. The panels show high resolution ARPES *E*(*k*_||_) dispersions measured at a temperature of *T* = 12 K and at a photon energy of h*ν* = 16 eV and 21 eV, respectively. In (**a**), the TSS, the bulk conduction band (BCB) and the bulk valence band (BVB) are indicated. (**c**) Longitudinal resistance *R*_xx_ vs perpendicular magnetic field *B* as symmetrized raw data measured at *T* = 0.47 K. Inset: Longitudinal resistance *R*_xx_ vs temperature *T* measured for *B* = 0. (**d**) Longitudinal resistivity *ρ*_xx_ (blue curve, left axis) and Hall resistivity *ρ*_xy_ (red curve, right axis) vs perpendicular magnetic field *B* measured at *T* = 0.47 K.

High-resolution ARPES dispersions measured at a temperature of 12 K for two representative photon energies of h*ν* = 16 eV and 21 eV are shown in Fig. 1a and b, respectively. We clearly observe distinct intensity contributions from the bulk conduction band (BCB) and bulk valence band (BVB) coexisting with sharp and intense Dirac cone representing the TSS. The BCB crossing the Fermi level indicates that the crystals are intrinsically *n*-type, in agreement with our Hall measurements on the same samples. At binding energies higher than the Dirac node (${E}_{{\rm{D}}}\sim 0.35$ eV), the lower half of the TSS overlaps with the BVB. By changing the photon energy we select the component of the electron wave vector perpendicular to the surface *k*_*z*_. Since the lattice constant of Bi_2_Se_3_ is very large along the *z* direction (*c* = 28.64 Å), the size of the bulk Brillouin zone (BBZ) is very small ($\sim 0.2$ Å^−1^). With photon energies between 16 to 21 eV we cross practically the complete BBZ, enhancing the sensitivity to the out-of-plane dispersion of the bulk bands. We note that the ARPES intensity changes with the photon energy as well due to the *k*_*z*_-dependence of the photoemission transitions. Differently from the BCB or BVB, the TSS exhibits no *k*_*z*_-dependence due to its 2D character. Consistent with the direct nature of the gap, we find the BCB minimum (≈Γ-point of the BBZ) at a binding energy of $\sim 0.154$ eV, while the BVB maximum is at $\sim 0.452$ eV. In particular, from the ARPES measurements, we estimate a bulk carrier density of *n*_3D,BCB_ = 1.77 ⋅ 10^19^ cm^−3^ and a sheet carrier density of ${n}_{\mathrm{2D},\mathrm{TSS}}={k}_{F,\mathrm{TSS}}^{2}\mathrm{/(4}\pi )=1.18\cdot {10}^{13}$ cm^−2^, with *k*_F,3D_ = 0.064 Å^−1^ and *k*_F,TSS_ = (0.086 ± 0.001) Å^−1^, respectively.

In the following, we present data measured on one Bi_2_Se_3_ macro flake. However, similar results were obtained for other samples from the same source bulk single crystal. The longitudinal resistance *R*_xx_ and the Hall resistance *R*_xy_ were measured simultaneously in a temperature range between 0.3 K and 72 K in tilted magnetic fields up to 33 T. *R*_xx_ as a function of perpendicular magnetic field *B* measured at *T* = 0.47 K is shown in Fig. 1c as symmetrized raw data ${R}_{{\rm{xx}}}^{{\rm{sym}}}(B)=[{R}_{{\rm{xx}}}^{{\rm{raw}}}(+B)+{R}_{{\rm{xx}}}^{{\rm{raw}}}(-B)]\mathrm{/2}$. The temperature-dependent *R*_xx_ at zero magnetic field shows metallic-like behavior (see inset of Fig. 1c). A residual resistance ratio RRR = *R*_xx_(288 K)/*R*_xx_(4.3 K) = 1.63 indicates a high crystalline quality^12^ (see Supplementary Information Sec. 1).

The longitudinal resistivity *ρ*_xx_ and the Hall resistivity *ρ*_xy_ as a function of the perpendicular magnetic field *B* at a temperature of *T* = 0.47 K are shown in Fig. 1d (*ρ*_xx_: blue curve, left axis; *ρ*_xy_: red curve, right axis). The onset of quantum oscillations with plateaux-like features in *ρ*_xy_ and SdH oscillations in *ρ*_xx_ can be observed at fields *B* ≥ 10 T. The low-field slope of *ρ*_xy_ yields a carrier density of *n*_Hall_ = 1.97 ⋅ 10^19^ cm^−3^ and a carrier mobility of *μ*_Hall_ = 594 cm^2^/(Vs).

**Figure 2:**
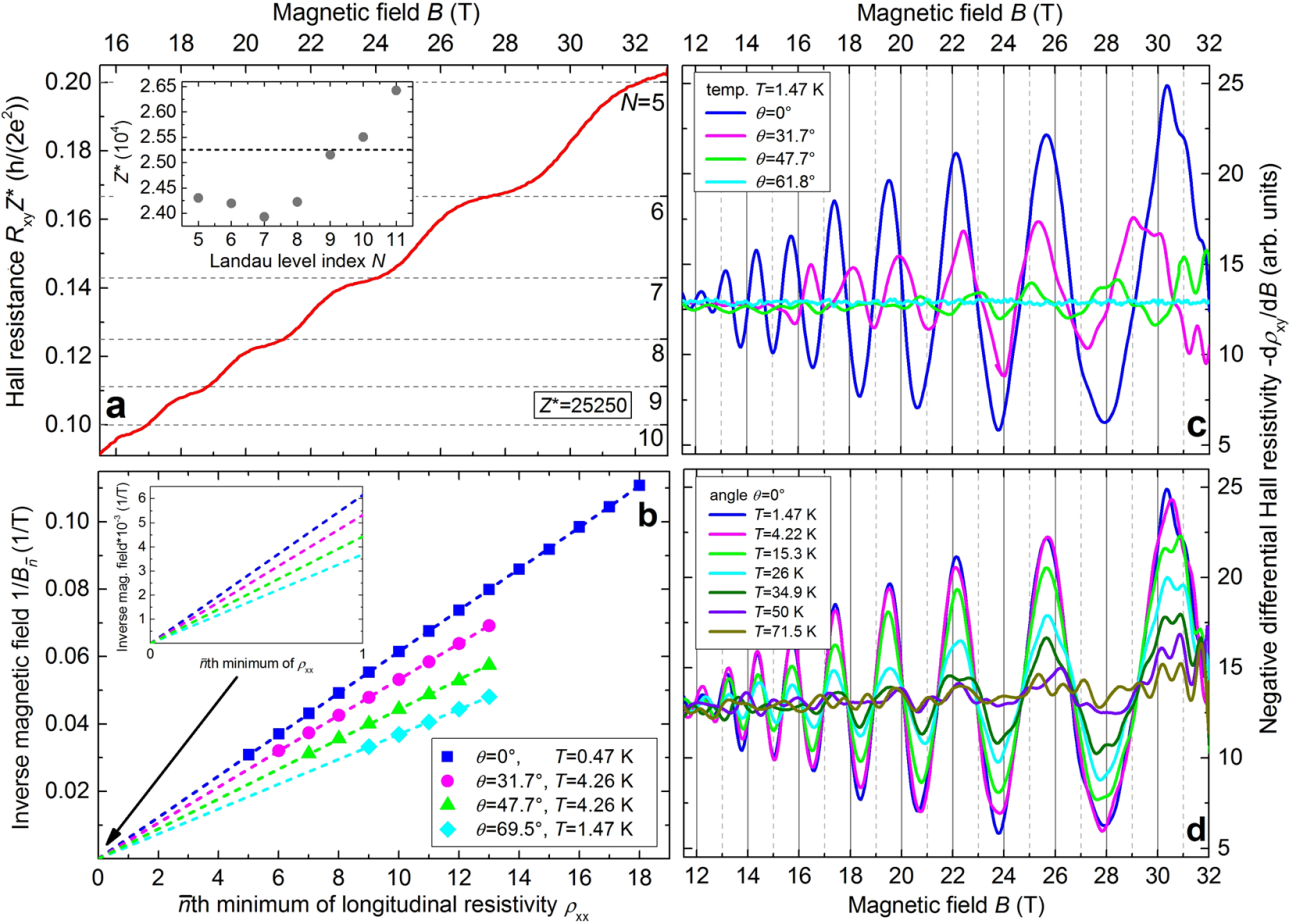
Quantum oscillations of the Hall resistance. (**a**) Hall resistance ${\tilde{R}}_{{\rm{xy}}}={R}_{{\rm{xy}}}{Z}^{\ast }$ in units of *h*/(2*e* ^2^) with an averaged number of 2D layers *Z* ^*^ = 25250 vs magnetic field *B* at *T* = 0.47 K and *θ* = 0°. Inset: Number of 2D layers *Z* ^*^ vs Landau level (LL) index *N*. The averaged number of 2D layers *Z* ^*^ = 25250 is shown as dashed line. (**b**) LL fan diagram from SdH oscillations of longitudinal resistivity *ρ*_xx_ for different values of the angle *θ* between the direction of the magnetic field $\overrightarrow{B}$ and the surface normal $\overrightarrow{N}$ of the Bi_2_Se_3_ macro flake and for different temperatures *T*. The dashed lines represent the best linear fits to the data. Inset: Enlargement of the LL fan diagram for $0\le \bar{n}\le 1$. (**c**) and (**d**) Negative differential Hall resistivity −d*ρ*_xy_/d*B* vs magnetic field *B*, for different values of the angle *θ* at *T* = 1.47 K and at different temperatures *T* for *θ* = 0°, respectively.

In order to analyze the plateaux-like features in *R*_xy_, we use the high-field anti-symmetrized Hall resistance ${R}_{{\rm{xy}}}^{{\rm{asy}}}(B)=[{R}_{{\rm{xy}}}^{{\rm{raw}}}(+B)-{R}_{{\rm{xy}}}^{{\rm{raw}}}(-B)]\mathrm{/2}$ data for *T* = 0.47 K and an angle of *θ* = 0°. *θ* denotes the angle between the direction of $\overrightarrow{B}$ and the surface normal $\overrightarrow{N}$ of the Bi_2_Se_3_ macro flake (i. e. *θ* = 0° means $\overrightarrow{B}$||$\overrightarrow{N}$). $\overrightarrow{N}$ is parallel to the *c*-axis of the single crystal. The scaling behaviour of ${\rm{\Delta }}{R}_{{\rm{xy}}}^{{\rm{asy}}}={R}_{{\rm{xy}}}^{{\rm{asy}}}(N)-{R}_{{\rm{xy}}}^{{\rm{asy}}}(N+\mathrm{1)}$ with the thickness leads to ${Z}^{\ast }=[\mathrm{(1/}N-\mathrm{1/(}N+\mathrm{1))/}{\rm{\Delta }}{R}_{{\rm{xy}}}^{{\rm{asy}}}]\cdot (h\mathrm{/(2}{e}^{2}))$ as the number of 2D spin-degenerate layers contributing to the transport. Conclusively, an average number of 2D layers of *Z* ^*^ = 25250 is derived. The variation of *Z* ^*^ for different Landau level (LL) index *N* is given in the inset of Fig. 2a.

**Figure 3:**
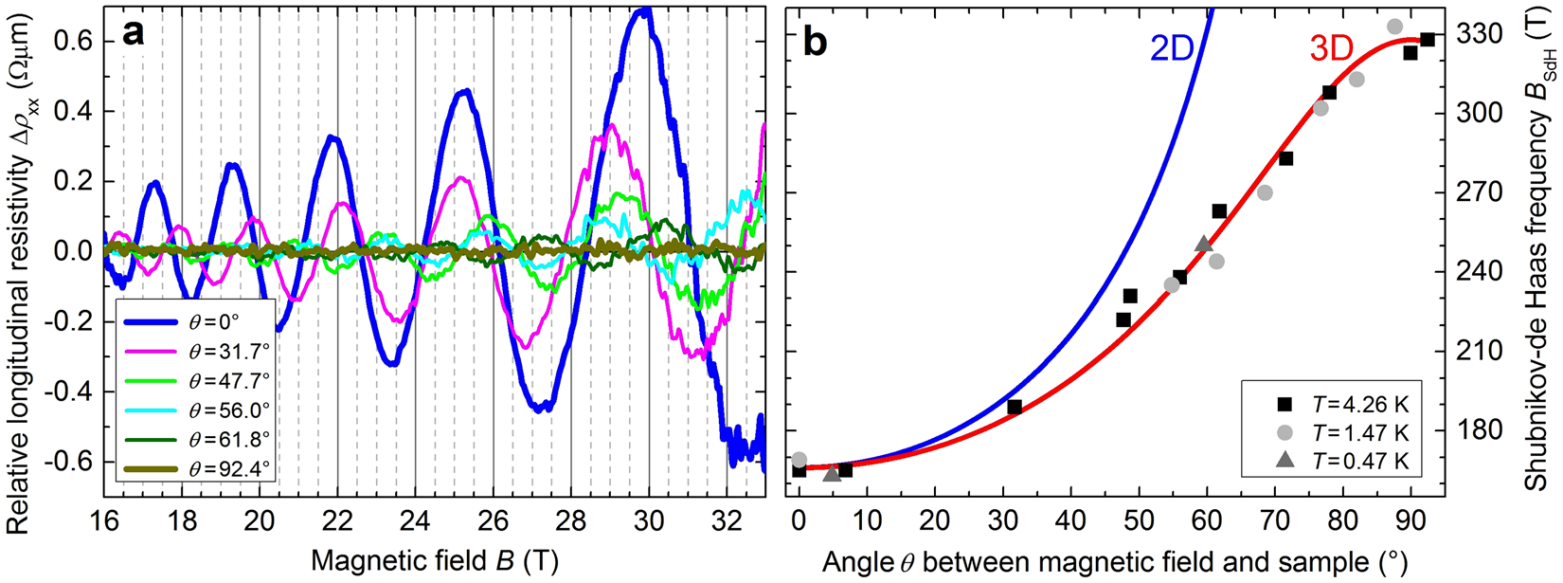
Angular-dependence of Shubnikov-de Haas oscillations. (**a**) Relative longitudinal resistivity Δ*ρ*_xx_ vs magnetic field *B* measured at *T* = 4.26 K for different values of the angle *θ*. (**b**) SdH frequency *B*_SdH_ vs angle *θ* determined at temperatures *T* = 0.47 K (dark gray triangles), 1.5 K (light gray circles), and 4.26 K (black squares). Curves represent calculated behavior for a planar 2D Fermi surface assuming ${B}_{{\rm{SdH}}}^{{\rm{2D}}}={B}_{\perp }/\,\cos \,\theta$ (blue curve) and for an ellipsoidal 3D Fermi surface assuming ${B}_{{\rm{SdH}}}^{{\rm{3D}}}={B}_{\perp }{B}_{||}/\sqrt{{({B}_{\perp }\cos \theta )}^{2}+{({B}_{||}\sin \theta )}^{2}}$ (red curve) with *B*_⊥_ = 166 T (for *θ* = 0°) and *B*_||_ = 328 T (for *θ* = 90°).

**Figure 4:**
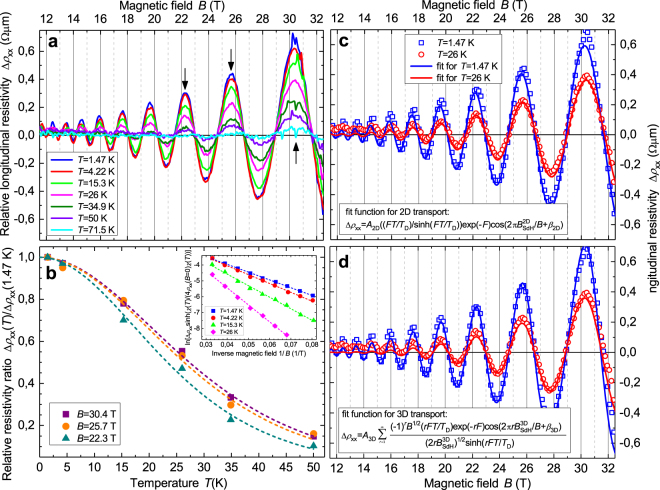
2D and 3D analysis of the temperature dependence of the Shubnikov-de Haas oscillations. (**a**) Relative longitudinal resistivity Δ*ρ*_xx_ vs magnetic field *B* measured for an angle *θ* = 0° at different temperatures *T*. The black arrows indicate values of magnetic field *B* shown in panel b vs temperature *T*. (**b**) Relative longitudinal resistivity ratio Δ*ρ*_xx_(*T*)/Δ*ρ*_xx_(1.47 K) vs temperature *T* for a magnetic field of *B* = 30.4 T (violet squares), 25.7 T (orange circles), and 22.3 T (dark cyan triangles). Dashed curves represent best fits to data assuming the function *χ*(*T*)/sinh(*χ*(*T*)), with *χ*(*T*) = (4*π* ^3^ *m* ^*^ *k*_B_ *T*)/(*heB*). Inset: Dingle plots of the SdH oscillations with 2D transport (maxima of relative longitudinal resistivity Δ*ρ*_xx_ as shown in panel (a)) at *T* = 1.47 K (blue squares), 4.22 K (pink circles), 15.3 K (green triangles), and 26 K (cyan diamonds). Dashed lines represent best linear fits to data with the function −*πm* ^*^/(*eτ*_D_ *B*), with *m* ^*^ = 0.16 *m*_e_. (**c**) Relative longitudinal resistivity Δ*ρ*_xx_ vs magnetic field measured for *θ* = 0° at *T* = 1.47 K (blue squares) and 26 K (pink circles). For clarity, only every fifth data point is shown. The curves are best fits to the Lifshitz-Kosevich formula for 2D transport (given in the legend), with ${B}_{{\rm{SdH}}}^{{\rm{2D}}}=166$ T (cf. Fig. 3b and text). (**d**) Same data as in (**c**): The curves are best fits to the Lifshitz-Kosevich formula for 3D transport (given in the legend), with ${B}_{{\rm{SdH}}}^{{\rm{3D}}}=169.5$ T and the parameter *F* = 2*π k*_B_ *T*_D_/($\bar{h}$ *ω*_C_) = *m* ^*^/(*τ*_D_ *eB*) and *m* ^*^ = 0.16 *m*_e_. The parameter *r* denotes the number of harmonic oscillations. In the present study we considered a range of values of 1 ≤ *r* ≤ 20.

The negative differentiated Hall resistivity −d*ρ*_xy_/d*B* vs magnetic field *B* is shown for different angles *θ* at constant *T*= 1.47 K in Fig. 2c, and for different temperatures *T* at constant *θ* = 0° in Fig. 2d. In accordance with the angular and the temperature dependence of the SdH oscillations, as shown in Figs 3 a and 4a, respectively, a decreasing amplitude of the differentiated Hall resistivity with increasing *θ* and increasing *T* is detected. At a constant *T* = 1.47 K the typical signatures of quantum oscillations of the Hall resistance are observed up to *θ* = 61.8° and at *θ* = 0° the amplitude of d*ρ*_xy_/d*B* vanishes only for temperatures above 71.5 K.

The SdH oscillations in the longitudinal resistivity *ρ*_xx_ are periodic functions of the inverse magnetic field 1/*B* and the minima correspond to an integer number of filled LLs. Since the degeneracy of states in the LLs is proportional to *B*, the inverse magnetic-field positions of the *ρ*_xx_ minima are linear functions of the LL index. The slopes of the linear functions depend on the extremal cross-sectional area of the Fermi sphere (for 3D systems) or circle (for 2D systems), and the intercepts depend on the Berry phase of the charge carriers (see Supplementary Information Sec. 2). In the LL fan diagram shown in Fig. 2b the inverse magnetic-field positions $\mathrm{1/}{B}_{\bar{n}}$ are plotted vs the $\bar{n}$th minimum of the longitudinal resistivity *ρ*_xx_ for different values of *θ*. The straight dashed lines, which represent the best linear fits to the data, intersect jointly the $\bar{n}$-axis at the point of origin (see Supplementary Information Sec. 2). Hence, we find for all angles *θ* and temperatures *T* investigated here a significant evidence for a trivial Berry phase of Φ_B_ = 0 (cf. inset of Fig. 2b) and conclude the dominance of non-relativistic fermions. For an improved estimate of the Berry phase we have fitted the behavior of the relative longitudinal resistivity Δ*ρ*_xx_ vs magnetic field *B* assuming 2D and 3D transport (cf. Fig. 4c and d, respectively).

The relative longitudinal resistivity Δ*ρ*_xx_ vs magnetic field *B* measured at *T* = 4.26 K for different angles *θ* is shown in Fig. 3a. Δ*ρ*_xx_ was calculated from the measured *ρ*_xx_ by subtracting a suitable polynominal fit to the background to extract the oscillatory component. The amplitude of the SdH oscillations decreases with increasing angle *θ*, and is really marginal for *θ* > 70°. Furthermore, a change in the frequency of the SdH oscillations with increasing angle *θ* with respect to *θ* = 0° is observed. For all values of *θ* and *T*, we found one value of the SdH frequency *B*_SdH_, deduced from the periodicity in the 1/*B* dependence. These values are in agreement with those determined from the slopes of the lines in the LL fan diagram (Fig. 2b) and from fast Fourier transforms of the same data. The absence of additional frequencies and beatings, as well as the angular dependence of *B*_SdH_ (see Fig. 3b), are significant evidence of a single 3D (non-spherical) Fermi surface (see Supplementary Information Sec. 3).

The temperature dependence of Δ*ρ*_xx_ is shown in Fig. 4a: the amplitude decreases with increasing temperature *T*, and oscillations are not observed for *T* > 71.5 K. From the fitting of the relative longitudinal resistivity ratio Δ*ρ*_xx_(*T*)/Δ*ρ*_xx_(*T* = 1.47 K), we deduce an effective mass of the charge carriers of *m* ^*^ ≅ 0.16 *m*_e_ (*m*_e_ = 9.10938356 ⋅ 10^−31^ kg denotes the electron rest mass) and a Fermi velocity of *v*_F_ = $\bar{h}$ *k*_F,3D_/*m* ^*^ = 0.46 ⋅ 10^6^ m/s, with *k*_F,3D_ = 0.064 Å^−1^.

In a first step we assumed 2D transport in accordance with some other investigations^14,15,26^. The Dingle plots (inset of Fig. 4b) at temperatures of *T* = 1.47 K, 4.22 K, 15.3 K, and 26 K yield the following Dingle scattering time (also known as single-particle relaxation time) *τ*_D_ and the Dingle temperature *T*_D_ = *h*/(4*π* ^2^ *k*_B_ *τ*_D_), assuming the fit function −*πm* ^*^/(*eτ*_D_ *B*) with *m* ^*^ = 0.16 *m*_e_: *τ*_D_ = 5.8 ⋅ 10^−14^ s (*T*_D_ = 20.8 K), 5.1 ⋅ 10^−14^ s (23.7 K), 3.9 ⋅ 10^−14^ s (30.9 K) and 2.7 ⋅ 10^−14^ s (45.5 K), respectively. For a more detailed analysis we have fitted the magnetic-field dependence of Δ*ρ*_xx_ (see Supplementary Information Sec. 4) and have used as fit function the Lifshitz-Kosevich formula^4,27,28^ for 2D transport. We found a reasonably good agreement between experimental data and the calculated behavior for Δ*ρ*_xx_(*B*) (cf. Fig. 4c).

However, in a second step we also performed fits under the assumption of 3D transport^29^ (cf. Fig. 4d), because the angular dependence of the SdH frequency *B*_SdH_ in Fig. 3b clearly follows the function for 3D transport. In this case, we find for all curves a single value for the Dingle temperature *T*_D_ = 23.5 K and hence a single value for the Dingle scattering time *τ*_D_ = 5.2 ⋅ 10^−14^ s, consistent with a nearly constant *R*_xx_(*T*) up to *T* = 30 K (see inset of Fig. 1c). From *τ*_D_ and the effective mass *m* ^*^ = 0.16 *m*_e_, we determined a carrier mobility of *μ*_D_ = *eτ*_D_/*m* ^*^ = 572 cm^2^/(Vs).

### Evaluation of experimental data

Most of the investigations of bulk Bi_2_Se_3_ conclude that the Fermi surface is 3D^3,12,14,17,30^, usually from the angular dependence of the SdH oscillations. However, in the search of TSS and QHE some works evaluated the Fermi surface as 2D^15,16^. Our analysis of the SdH oscillations (see above) indicates that the Fermi surface is 3D. This is confirmed by our following analysis of the angular dependence of the SdH frequencies.

The angle dependence of the SdH oscillations determines that the Fermi surface has an ellipsoidal shape. For a plane 2D Fermi surface, the SdH oscillation frequency is equal to ${B}_{{\rm{SdH}}}^{{\rm{2D}}}(\theta )={B}_{\perp }/\,\cos \,\theta$, with ${B}_{{\rm{SdH}}}^{{\rm{2D}}}(\theta )\to \infty$ for *θ* → 90° (blue curve in Fig. 3b), and for an ellipsoidal 3D Fermi surface it is ${B}_{{\rm{SdH}}}^{{\rm{3D}}}(\theta ))={B}_{\perp }{B}_{||}/\sqrt{{({B}_{||}\cos \theta )}^{2}+{({B}_{\perp }\sin \theta )}^{2}}$ (red curve in Fig. 3b), with ${B}_{\perp }={B}_{{\rm{SdH}}}^{{\rm{3D}}}(\theta ={0}^{\circ })={B}_{{\rm{SdH}}}^{{\rm{2D}}}(\theta ={0}^{\circ })=166$ T and ${B}_{||}={B}_{{\rm{SdH}}}^{{\rm{3D}}}(\theta ={90}^{\circ })=328$ T. Previous data^15,16^ may also be interpreted as 3D ellipsoidal Fermi surface (see Supplementary Information Sec. 3).

We estimate the ellipsoidal cross-section of the 3D Fermi surface with the wave vectors ${k}_{F,\mathrm{SdH}}^{(a)}={k}_{F,\mathrm{SdH}}^{(b)}=$ $\sqrt{2e{B}_{\perp }/\hslash }=0.071$ Å^−1^ and ${k}_{F,\mathrm{SdH}}^{(c)}=2e{B}_{||}/(\hslash {k}_{F,\mathrm{SdH}}^{(a)})=0.14$ Å^−1^. With these values we deduced an eccentricity for the 3D non-spherical Fermi surface of ${k}_{F,\mathrm{SdH}}^{(c)}/{k}_{F,\mathrm{SdH}}^{(a)}=1.98$. Köhler^30^ and Hyde *et al*.^12^ show, that the eccentricity of the Fermi surface decreases with decreasing carrier density *n*. In accordance with the present study, Eto *et al*.^14^ deduced for a Bi_2_Se_3_ bulk single crystal with a lower carrier density of *n* = 3.4 ⋅ 10^18^ cm^−3^ an eccentricity of ${k}_{F,\mathrm{SdH}}^{(c)}/{k}_{F,\mathrm{SdH}}^{(a)}=1.62$, consistent with eccentricities obtained by Köhler^30^. Assuming a parabolic dispersion and using the values of ${k}_{{\rm{F}}}^{(a)}$ and ${k}_{{\rm{F}}}^{(c)}$ from the SdH analysis and of *E*_F_ from the ARPES measurements, we estimate with *E*_F_ = ($\bar{h}$ *k*_F_)^2^/(2 *m* ^*^) for the effective masses ${m}_{{\rm{a}}}^{\ast }={m}_{{\rm{b}}}^{\ast }=0.125\,{m}_{{\rm{e}}}$ and ${m}_{{\rm{c}}}^{\ast }=0.485\,{m}_{{\rm{e}}}$. An average value for the effective mass is then given by^31^ $\mathrm{1/}{m}^{\ast }=\mathrm{(1/}{m}_{{\rm{c}}}^{\ast }+\mathrm{2/}{m}_{{\rm{a}}}^{\ast }\mathrm{)/3}$, which yields *m* ^*^ = 0.166 *m*_e_. This value is consistent with the value obtained from the temperature dependence of the SdH oscillations: ${m}_{{\rm{SdH}}}^{\ast }=0.16{m}_{{\rm{e}}}$.

## Discussion

Generally, a bulk or 3D QHE is attributed to parallel 2D conduction channels, each made from one or a few stacking layers. A bulk QHE, where quantized values of the Hall resistance *R*_xy_ inversely scale with the sample thickness, has been observed in a number of anisotropic, layered electronic bulk materials, e.g., GaAs/AlGaAs multi-quantum wells^20^, Bechgaard salts^24,32^ and also in Fe-doped Bi_2_Se_3_ bulk samples^33^, where transport by TSS was excluded. However, the observation of the quantum oscillations of the Hall resistance in Bi_2_Se_3_ at elevated temperatures calls for a special condition considering the usual requirement of $\mu B\gg 1$. In the present case *B*_max_ = 33 T and the carrier mobility *μ* ≈ 600 cm^2^/(Vs) yields only *μB*_max_ ≈ 2. Furthermore, the deduced effective mass *m* ^*^ = 0.16 *m*_e_ yields for a magnetic field of *B* = 10 T, where we observe the onset of the quantum oscillations, a value for the LL energy splitting of $\bar{h}$ *ω*_c_ = $\bar{h}$ *eB*/*m* ^*^ ≈ 7 meV. However, the thermal energy amounts to *k*_B_ *T* ≈ 4 meV at *T* = 50 K, while $\hslash {\omega }_{{\rm{c}}}\gg {k}_{{\rm{B}}}T$ is usually required for a QHE. Nevertheless, we observe unambiguous quantum oscillations in −d*ρ*_xy_/d*B* (Fig. 2c and d) as signature of a QHE.

In order to explain the experimental observations, we propose the following model. The Bi_2_Se_3_ bulk sample investigated here may consist of three different conducting regions: a semiconducting-like core region, surrounded by a metallic-like shell region and the topological surface (see Fig. S1 in Supplementary Information Sec. 5). The semiconducting-like core was proven by the preparation of semiconducting micro flakes^3^. The metallic-like shell region due to Se depletion dominates the transport mechanism observed here as metallic and 2D layered effects. From our experiments, we assume the shell to form a stacked system of 2D layers with a periodic potential^23^ either due to the van der Waals-gaps or the unit cell along the *c*-axis because of the carrier density modulation due to Se vacancies. In magnetic fields *B* ≠ 0 the thickness scaling of the plateaux-like features in the Hall resistance yields an effective thickness for the shell of stacked 2D layers. For the charge carrier density, we estimate three different values for the core (from ref.^3^), the shell (from the Hall measurements) and the topological surface (from the ARPES measurements): *n*_core_ ≈ 1.2 ⋅ 10^17^ cm^−3^, *n*_shell_ ≈ 2 ⋅ 10^19^ cm^−3^ and *n*_TSS_ = 1.2 ⋅ 10^13^ cm^−2^, respectively. In the semiconducting-like core region, the Fermi level (chemical potential) is in the gap close to the bottom of the conduction band, whereas in the metallic-like shell region the Fermi level is in the conduction band (see Supplementary Information Sec. 5). Because of a finite scattering rate between the 2D layers in the shell region *ρ*_xx_(*B*) shows considerable 3D character in the SdH oscillations for higher angles *θ*, and the quantization in *ρ*_xy_(*B*) even at the lowest temperature *T* is not exact and the plateaux have a finite slope.

For the SdH frequency *B*_SdH_ we estimate at *θ* = 0° for the three regions the following values: *B*_SdH,core_ = 4.82 T, *B*_SdH,shell_ = 166 T and *B*_SdH,TSS_ = 248 T. The small value *B*_SdH,core_ corresponds to a slow-changing background which is out of the measurement range of our experimental setup. The larger value of the TSS is caused only by the small number of surface electrons with respect to the large number of bulk electrons (*N*_bulk_ ≈ *N*_shell_ ≈ 2 ⋅ 10^15^ and *N*_TSS_ ≈ 3 ⋅ 10^11^ yield a ratio *N*_TSS_/*N*_bulk_ ≈ 10^−4^). Therefore, from the experimental data we deduce only the *B*_SdH_ value for the shell (see Fig. 3a) and find the dominant contribution of the bulk (core + shell) in the transport behavior. A periodic modulation of the charge carrier density along the *c*-direction would result in a miniband structure for the LLs and, as long as the Fermi level is in a gap between these minibands, the Hall resistivity *ρ*_xy_ will be quantized and scale with the periodicity of the potential^23^.

According to our estimate of the width of the LLs (see above), the persistence of the quantum oscillations in the Hall resistance up to high temperatures requires a special condition: We propose a Fermi level pinning in the miniband gap, which could be the result of an interaction with the existing TSS. Theoretically, due to the inter-layer coupling, it is expected that in the quantum Hall state the edge states of the stacked 2D layers form a sheath at the surface^34^. Due to the finite width of the wave functions at the surface, this sheath can interact with the TSS. This opens the possibility that the TSS act as electron reservoirs to pin the Fermi level in a miniband gap as the magnetic field is varied over a finite range. Therefore, we conclude that the observation of the quantum oscillations of the Hall resistance at higher temperatures in Bi_2_Se_3_ (*n* ≈ 2 ⋅ 10^19^ cm^−3^) with a majority of non-Dirac fermions is related to the existence of the TSS. Based on our results, we propose that other 3D materials with TSS and a periodic potential modulation may show quantization effects in the Hall resistance at elevated temperatures.

## Methods

High-quality single crystalline Bi_2_Se_3_ was prepared from melt with the Bridgman technique. The growth time, including cooling was about 2 weeks for a ∼50 g crystal. The whole crystal was easily cleaved along the [00.1] growth direction, indicating crystal perfection. The macro flake was prepared by cleaving the bulk single crystal with a thickness of around 110 *μ*m to investigate bulk properties.

We explored the structural properties of the bulk single crystal^3^ with atomic force microscopy (AFM), scanning transmission electron microscopy (STEM) and high-resolution transmission electron microscopy (HRTEM). The composition and surface stability were investigated using energy-dispersive x-ray spectroscopy (EDX) and spatially resolved core-level X-ray PEEM. Structural analysis using HRTEM and STEM was carried out at a JEOL JEM2200FS microscope operated at 200 kV. The sample preparation for HRTEM characterization consisted of ultrasonic separation of the flakes from the substrate, followed by their transfer onto a carbon-coated copper grid. Using adhesive tape, the surface was prepared by cleavage of the crystal along its trigonal axis in the direction perpendicular to the van-der-Waals-type (0001) planes. The ARPES measurements were performed at a temperature of 12 K in an ultra-high vacuum (UHV) chamber at a pressure of $\sim 5\cdot {10}^{-10}$ mbar with a VG Scienta R8000 electron analyzer at the UE112-PGM2a beamline of BESSY II using p-polarized undulator radiation.

Magnetotransport experiments were performed using standard low-noise lock-in techniques (Stanford Research Systems SR830 with a Keithley 6221 as current source), with low excitation to prevent heating of the sample. The Bi_2_Se_3_ macro flake was mounted in a flow cryostat (1.3 K to 300 K), as well as in a ^3^He insert (down to 0.3 K), in a Bitter magnet with a bore diameter of 32 mm and magnetic fields up to 33 T at the High Field Magnet Laboratory of the Radboud University Nijmegen. In both setups, a Cernox thermometer in the vicinity of the sample was used to monitor the temperature *in situ*. In the ^3^He system, the temperature between 0.3 K and 1.3 K was stabilized by the ^3^He vapour pressure prior to the magnetic field sweep to assure a constant temperature. However, the temperature between 1.3 K and 4.2 K was stabilized by the ^4^He pressure. Above a temperature of 4.2 K, we have used the flow cryostat and stabilized the temperature using a capacitance.

## Electronic supplementary material

Supplementary Information
