# Supplementary material for: High-temperature quantum oscillations of the Hall resistance in bulk Bi2Se3
Source: Sci Rep. 2018 Jan 11;8:485. doi: 10.1038/s41598-017-18960-0 (PMC5764972; doi:10.1038/s41598-017-18960-0)
Supplement: Supplementary file 1 — Supplementary Information [file 41598_2017_18960_MOESM1_ESM.pdf]

Supplementary Information: High-temperature quantum oscillations of the Hall resistance in bulk Bi_2Se_3

Marco Busch,[1] Olivio Chiatti,[1] Sergio Pezzini,[2] Steffen Wiedmann,[2] Jaime Sánchez-Barriga,[3] Oliver Rader,[3] Lada V. Yashina,[4] and Saskia F. Fischer[1]

[1]*Novel Materials Group, Humboldt-Universität zu Berlin, Newtonstraße 15, 12489 Berlin, Germany*
[2]*High Field Magnet Laboratory, Radboud University Nijmegen, P.O. box 9010, 6500 GL Nijmegen, Netherlands*
[3]*Helmholtz-Zentrum-Berlin für Materialien und Energie, Albert-Einstein-Straße 15, 12489 Berlin, Germany*
[4]*Department of Chemistry, Moscow State University, Leninskie Gory 1/3, 119991 Moscow, Russia*
(Dated: December 8, 2017)

1. Discussion of the residual resistance ratio

The residual resistance ratio $\mathrm{RRR} = R_{\mathrm{xx}}(288\ \mathrm{K})/R_{\mathrm{xx}}(4.3\ \mathrm{K}) = 1.63$ deduced in the present study is in accordance with $\mathrm{RRR} = \rho_{\mathrm{xx}}(300\ \mathrm{K})/\rho_{\mathrm{xx}}(2\ \mathrm{K}) = 1.74$, which we deduced in our previous study[1], as well as with the values of $\mathrm{RRR}=\rho_{\mathrm{xx}}(292\ \mathrm{K})/\rho_{\mathrm{xx}}(5\ \mathrm{K})=1.63$ and $\mathrm{RRR}=\rho_{\mathrm{xx}}(300\ \mathrm{K})/\rho_{\mathrm{xx}}(2\ \mathrm{K})=1.47$ deduced by Cao *et al.*[2] and Petrushevsky *et al.*[3], respectively. For single crystalline Bi_2Se_3 with a six times smaller carrier density of $n_{\mathrm{Hall}}=3.4\cdot10^{18}\ \mathrm{cm}^{-3}$ and an eleven times larger carrier mobility of $\mu_{\mathrm{Hall}}=6500\ \mathrm{cm}^2/(\mathrm{Vs})$, Eto *et al.*[4] found a two-times larger residual resistivity ratio of $\mathrm{RRR} = \rho_{\mathrm{xx}}(300\ \mathrm{K})/\rho_{\mathrm{xx}}(1.5\ \mathrm{K}) = 0.9\ \mathrm{m\Omega cm}/(0.28\ \mathrm{m\Omega cm}) = 3.2$. A larger value of RRR of about 2.7 with respect to our result was found by Kumar & Lakhani[5] on Bi_2Se_3 microstrained single crystals with a carrier density of $n_{\mathrm{Hall}}=1.13\cdot10^{19}\ \mathrm{cm}^{-3}$. The microstrain arises from enhanced crystalline defects (e.g. vacancies and dislocations) and produce an inhomogeneous local strain field which creates the regions of vanishing and non-vanishing bulk band gap.

2. Determination of the Berry Phase from the Landau level fan diagram

Fig. 2b shows the Landau level (LL) fan diagram determined for different temperatures T and different values of the angle θ between the direction of the magnetic field $\vec{B}$ and the surface normal $\vec{N}$, which is collinear to the c-axis of the Bi_2Se_3 macro flake. Here and in the following, an angle of $\theta=0°$ means a perpendicular magnetic field and an angle of $\theta = 90°$ means a parallel magnetic field with respect to the surface plane of the Bi_2Se_3 macro flake. The $1/B$-positions of the SdH-minima of the longitudinal resistance R_{xx} curves are shown in Fig. 2b as a function of the corresponding LL indices. The LL index N of the SdH oscillations is related to the extremal cross-section area $\tilde{A}=\pi k_{\mathrm{F}}^2$ of the Fermi surface, given by the Fermi wave vector k_{F} perpendicular to the magnetic field B through the semiclassical Lifshitz-Onsager equation[6–14]:

$$2\pi(N+\gamma) = \pi k_{\mathrm{F}}^2 \frac{\hbar}{eB} \tag{1}$$

This relation holds when the Nth Landau level is crossing the Fermi energy E_{F}. The parameter γ is the phase factor of the SdH oscillation and is generally given by[11,14,15]:

$$\gamma = 0.5 - \Phi_{\mathrm{B}}/(2\pi) - \delta. \tag{2}$$

The Berry phase[16] Φ_{B} is equal zero for an electronic system with non-relativistic fermions (Schrödinger electrons) with parabolic energy-momentum dispersion, as in the case of conventional metals for example[17–19]. In contrast, a non-trivial Berry phase of $\Phi_{\mathrm{B}}=\pi$ reflects the existence of a singularity in the energy bands, e.g. a band-contact line in 3D bulk states or a Dirac point in 2D surface states such as in graphene[20–23], bulk Rashba semiconductors such as BiTeI[14], in Weyl semimetals such as TaAs[24], TaP[25] and NbP[26] or in Dirac semimetals such as Cd_3As_2[27] and $ZrTe_5$[28]. In TI materials, the energy-momentum dispersion is not strictly linear as in the case of massless Dirac fermions, but contains a parabolic component. Nevertheless, theoretically it has been elucidated that the Berry phase Φ_{B} is equal to π at least at a large LL index N[12,13,29].

The parameter δ in the Eq. (2) denotes the phase shift correction determined by the dimensionality of the Fermi surface, with $\delta=0$ for a plane 2D Fermi surface and $\delta=\pm0.125$ for an ellipsoidal 3D Fermi surface (+ for electrons and − for holes)[11,14,15]. By neglecting the effect of the Zeeman splitting, which is significant only for the lower Landau levels in the quantum limit[12], $\gamma=0.5$ for a non-relativistic fermion system (Berry phase $\Phi_{\mathrm{B}}=0$) with a 2D Fermi surface and $\gamma=0.375$ (with $\delta=+0.125$ for electrons) in the case of a 3D Fermi surface, respectively. For a non-trivial Berry phase $\Phi_{\mathrm{B}}=\pi$ in the case of a system with linear energy-momentum dispersion, one has to distinguish between $\gamma=0$ for 2D Dirac fermions and $\gamma=\mp0.125$ for 3D Dirac fermions (− for electrons and + for holes). We will note

here, that in the literature[3,6,12,30–32] the phase factor γ is also defined as $\gamma = 0.5 - \Phi_B/(2\pi)$ neglecting the phase shift correction δ.

Experimentally, the phase factor γ can be obtained from the analysis of the Landau level (LL) fan diagram. The most straightforward method to plot such a LL fan diagram is to assign an integer number $\bar{n}$ to the minima and/or a half integer number $\hat{n}$ to the maxima of the longitudinal resistivity ρ_{xx}, which is the general convention[6,12]. Then, the $\hat{n}$th maximum in ρ_{xx} occurs at an inverse magnetic field $1/B_{\hat{n}}$ when (cf. Eq. (1)):

$$2\pi\left(\frac{\hbar k_F^2}{2eB_{\hat{n}}} - \gamma\right) = 2\pi\hat{n}, \tag{3}$$

and the $\bar{n}$th minimum in ρ_{xx} occurs at $1/B_{\bar{n}}$ when:

$$2\pi\left(\frac{\hbar k_F^2}{2eB_{\bar{n}}} - \gamma\right) = 2\pi\left(\bar{n} - \frac{1}{2}\right). \tag{4}$$

Extrapolating the linear relation to the origin yields the phase factor γ as the interecept with the horizontal axis.

From the evaluation of the LL fan diagram (see Fig. 2b) we deduced in accordance with other works[4,6,12] a trivial Berry phase of $\Phi_B = 0$, which indicates non-relativistic fermions. Eto *et al.*[4] have plotted in a LL fan diagram the positions of the maxima in the longitudinal resistivity ρ_{xx} as function of the LL index for different angles θ. From the fitted straight lines which intersect the horizontal axis at the same point $\neq 0$, they deduced a Berry phase of $\Phi_B \approx 0$ close to the exact value 0 for non-relativistic fermions. Cao *et al.*[2] obtain the same result in their LL fan diagram.

3. Angular dependence of the Shubnikov de Haas oscillations

We have plotted in Fig. 3b the values of the SdH frequency B_{SdH} vs angle θ for different temperatures and the calculated behavior for a planar 2D Fermi surface, assuming $B_{SdH}^{2D} = B_\perp/\cos\theta$ (blue curve) with $B_{SdH}^{2D} \to \infty$ for $\theta \to 90°$, and for an ellipsoidal 3D Fermi surface, assuming $B_{SdH}^{3D} = B_\perp B_{||}/\sqrt{(B_{||}\cos\theta)^2 + (B_\perp \sin\theta)^2}$ (red curve) with $B_\perp = B_{SdH}^{3D}(\theta = 0°) = B_{SdH}^{2D}(\theta = 0°) = 166$ T and $B_{||} = B_{SdH}^{3D}(\theta = 90°) = 328$ T. For all values of the angle θ and temperature T investigated in the present study, we found only one value of the SdH frequency B_{SdH} for every angle θ.

Our result is in strong contrast with the result of Petrushevsky *et al.*[3]. These authors concluded a plane 2D Fermi surface for Bi_2Se_3 bulk single crystals with a carrier density of $n_{Hall} = 5.6 \cdot 10^{19}$ cm^{-3} and a carrier mobility of $\mu_{Hall} = 400$ cm^2/(Vs). For the angle θ in the range $0° \leq \theta \leq 75°$ Petrushevsky *et al.*[3] deduced a SdH frequency B_{SdH} in the range 200 T $\leq B_{SdH} \leq$ 700 T. However, if we assume that $B_{SdH}(\theta)$ follows the 3D formula, the data of Petrushevsky *et al.*[3] would be consistent with an ellipsoidal 3D Fermi surface with an eccentricity of $k_F^{(c)}/k_F^{(a)} \approx 5$. This value is consistent with Hyde *et al.*[33] for a carrier concentration of $n \approx 6 \cdot 10^{19}$ cm^{-3} (with a Fermi energy of $E_F \approx 360$ meV) and indicates that the SdH data of Petrushevsky *et al.*[3] could be interpreted as 3D Fermi surface. Cao *et al.*[2] derived from the evaluation of the minima positions B_{min} in the longitudinal resistance R_{xx} a dependence $B_{min}(\theta) = B_{min}(\theta = 0°)/\cos\theta$ for the investigated angular range $0° \leq \theta \leq 43°$ and have also concluded a 2D-like transport. If our data are considered only in the range $0° \ll \theta \ll 43°$, a $1/\cos\theta$ dependence could be fitted approximately.

4. Temperature dependence of the Shubnikov de Haas oscillations

We have fitted (cf. Fig. 4b) the temperature dependence of the SdH oscillation amplitude at a perpendicular magnetic field ($\theta = 0°$) of $B = 30.4$ T (violet squares), 25.7 T (orange circles), and 22.3 T (dark cyan triangles) to the Lifshitz-Kosevich formula[6,9,12,34,35] using the function:

$$\frac{\Delta\rho_{xx}(T)}{\Delta\rho_{xx}(1.47\text{ K})} = \frac{\chi(T)}{\sinh(\chi(T))} = \frac{4\pi^3 m^* k_B T/(heB)}{\sinh[4\pi^3 m^* k_B T/(heB)]} \tag{5}$$

The effective mass of the charge carriers m^* is the single fitting parameter in the present analysis. We deduced an averaged value of $m^* \cong 0.16 m_e$ and a Fermi velocity of $v_F = \hbar k_{F,3D}/m^* = 0.46 \cdot 10^6$ m/s with the bulk Fermi wave vektor $k_{F,3D} = 0.064$ Å^{-1} deduced from our ARPES measurements (see text above).

For a more detailed analysis we have fitted the magnetic-field dependence of the relative longitudinal resistivity $\Delta\rho_{xx}$. In a first step we assumed 2D transport in accordance with other investigations[3,4,30,32] and have used as fit function the Lifshitz-Kosevich formula[6,9,12,34,35]:

$$\Delta\rho_{xx} = A_{2D}\rho_{xx}(B = 0)\exp(-\pi/(\mu B))\cos(2\pi B_{SdH}^{2D}/B + \beta_{2D}) \tag{6}$$

The parameter A_{2D} denotes an amplitude matching factor, $\rho_{xx}(B=0)=5.4\ \Omega\mu$m the longitudinal resistivity at zero magnetic field, μ the carrier mobility, and $B_{SdH}^{2D}=166$ T the SdH frequency (cf. Fig. 3b). For the phase $\beta_{2D}=2\pi\gamma_{2D}$ we deduced for both temperatures a value of π, which is equivalent to a phase factor $\gamma_{2D}=0.5$ and corresponds to a trivial Berry phase $\Phi_B=0$. This finding agrees with the result from the evaluation of the LL fan diagram (cf. Fig. 1d). The deduced values of the amplitude matching factor A_{2D} and the carrier mobility μ are: $A_{2D}=0.88$ and $\mu=500$ cm^2/(Vs) for $T=1.47$ K, as well as $A_{2D}=1.11$ and $\mu=367$ cm^2/(Vs) for $T=26$ K.

Although, we found a reasonably good agreement between experimental data and the calculated behavior for $\Delta\rho_{xx}(B)$ under the assumption of 2D transport, we also performed fits under the assumption of 3D transport. This is motivated by the analysis of the angular dependence of the Shubnikov-de Haas frequency B_{SdH} shown in Fig. 3b, were we found a significant evidence for an ellipsoidal 3D Fermi surface. To fit the magnetic-field dependence of the relative longitudinal resistivity $\Delta\rho_{xx}$ assuming 3D transport, we employed the formula[36]:

$$\Delta\rho_{xx}=A_{3D}\sum_{r=1}^{\infty}\frac{(-1)^r\sqrt{B}(rFT/T_D)\exp(-rF)\cos(2\pi rB_{SdH}^{3D}/B+\beta_{3D})}{\sqrt{2rB_{SdH}^{3D}}\sinh(rFT/T_D)} \tag{7}$$

with the SdH frequency $B_{SdH}^{3D}=169.5$ T and the parameter $F=2\pi k_B T_D/(\hbar\omega_C)=rm^*/(\tau_D eB)$ with $m^*=0.16m_e$. The parameter r denotes the number of harmonic oscillations. In the present study we considered a range of values of $1\leq r\leq 20$. For the phase $\beta_{3D}=2\pi\gamma_{3D}$ we deduced for both temperatures a value of $3\pi/4$, which is equivalent to a phase factor $\gamma_{3D}=0.375=0.5-0.125$ and corresponds to a trivial Berry phase $\Phi_B=0$. The amplitude factor A_{3D} has a constant value of $2.5\cdot\rho_{xx}(B=0)=13.5\ \Omega\mu$m, which is defined by the longitudinal resistivity $\rho_{xx}(B=0)$ at zero magnetic field[36]. For the Dingle scattering time τ_D and the Dingle temperature T_D we deduced $\tau_D=5.2\cdot 10^{-14}$ s and $T_D=23.5$ K, respectively, for both temperatures and found good agreement between the exeperimental data and the calculated behavior of the relative longitudinal resistivity $\Delta\rho_{xx}(B)$ (cf. Fig. 4d). From τ_D and $m^*=0.16m_e$ we determined a carrier mobility of $\mu=e\tau_D/m^*=572$ cm^2/(Vs). This value is in agreement with the value $\mu_{Hall}=600$ cm^2/(Vs) deduced from the slope of the Hall resistivity ρ_{xy} (see text above).

5. Proposed model for investigated Bi_2Se_3 bulk sample

We propose that the Bi_2Se_3 bulk sample consists of three different regions (see Fig. 1a): a core region with semiconducting-like transport properties; a shell region with metallic-like transport properties; the topological surface. The core and the shell region have comparable size and form the bulk of the sample, and are surrounded by the topological surface. In the semiconducting-like core (white in Fig. 1a) the charge carrier density[1] is $n_{core}\approx 1.2\cdot 10^{17}$ cm^{-3} and the Fermi level (chemical potential) is near the bottom of the conduction band. In the metallic-like shell (shaded with parallel lines in Fig. 1a) the density is $n_{shell}\approx 2\cdot 10^{19}$ cm^{-3} and the Fermi level (chemical potential) is in the conduction band at the Fermi energy $E_F\approx 0.15$ eV. The shell consists of a stack of 2D layers with a periodic potential along the c-axis due to the Van der Waals gaps. Due to Se vacancies the average charge carrier density is much higher than in the core and is modulated by the periodic potential. In the equilibrium the electrochemical potential $\mu^*=\mu(x)-e\phi(x)$ (with the position-dependent chemical potential $\mu(x)$ and the position-dependent electric potential $\phi(x)$) is constant throughout the bulk (see Fig. 1b), However, there is a potential difference $\Delta\phi\approx 0.15$ V between the core and the shell. If we consider the interface between core and shell as a Schottky contact, the spacer layer thickness is given by[37]:

$$\Delta x=\sqrt{\frac{2\epsilon_r\epsilon_0\Delta\phi}{eN_D}} \tag{8}$$

Using the approximation $N_D\approx n_{shell}$ for the donator density and with the relative permittivity[33] $\epsilon_r\approx 15$ (ϵ_0 denotes the absolute permittivity) we obtain a spacer layer thickness of $\Delta x\approx 46$ nm.

---

[1] Chiatti, O. *et al.* 2D layered transport properties from topological insulator Bi_2Se_3 single crystals and micro flakes. *Sci. Rep.* **6**, 27483 (2016).

[2] Cao, H. *et al.* Quantized Hall Effect and Shubnikov-de Haas Oscillations in Highly Doped Bi_2Se_3: Evidence for Layered Transport of Bulk Carriers. *Phys. Rev. Lett.* **108**, 216803 (2012).

[3] Petrushevsky, M. *et al.* Probing the surface states in Bi_2Se_3 using the Shubnikov-de Haas effect. *Phys. Rev. B* **86**, 045131 (2012).

[4] Eto, K. Ren, Z., Taskin, A. A., Segawa, K. & Ando Y. Angular-dependent oscillations of the magnetoresistance in Bi_2Se_3 due to the three-dimensional bulk Fermi surface. *Phys. Rev. B* **81**, 195309 (2010).

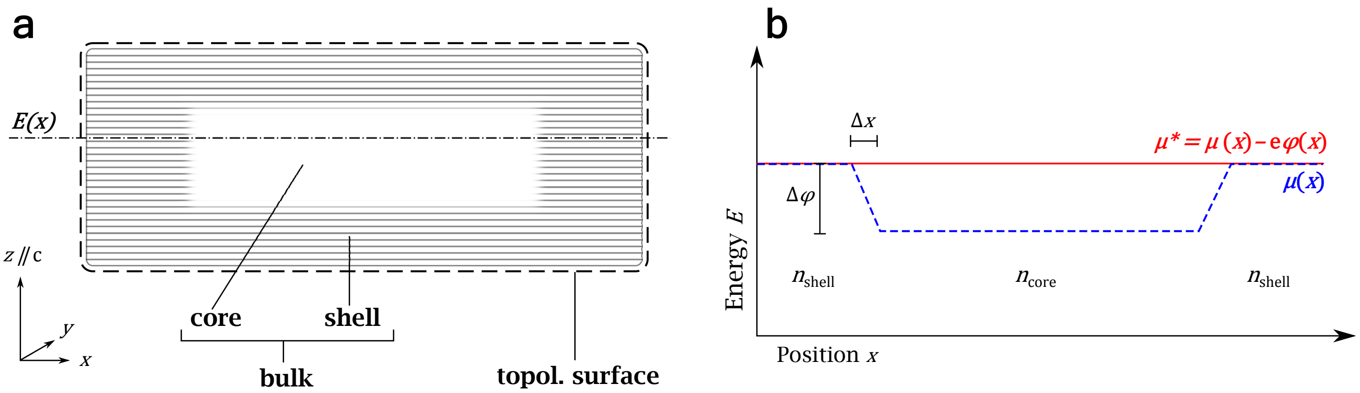

FIG. 1: **Proposed model for investigated Bi_2Se_3 bulk sample. a)**, The sample consists of three regions: a semiconducting-like core (white), surrounded by a metalic-like shell (shaded with parallel lines), surrounded itself by the topological surface (dashed line). The dashed-dotted line $E(x)$ is a cross-section of the electron energy vs position. **b)**, Electron energy vs position in th corss-section shown in a. $\mu(x)$ is the position-dependent Fermi level (chemical potential, blue), due to the different charge carrier density in the core and the shell. μ^* is the electrochemical potential (red), constant throughout the sample in equilibrium. $\phi(x)$ is the position-dependent electric potential. The interface between core and shell can be regarded as Schottky contact, with $\Delta\phi$ the barrier height and Δx the spacer layer thickness

[5] Kumar, D. & Lakhani, A. Observation of π Berry phase in quantum oscillations of three-dimensional Fermi surface in topological insulator Bi_2Se_3. *Phys. Status Solidi RRL* **9**, 636 (2015).
[6] Ando, Y. Topological Insulator Materials. *J. Phys. Soc. J.* **82**, 102001 (2013).
[7] Xiong, J. *et al.* High-field Shubnikov-de Haas oscillations in the topological insulator Bi_2Te_2Se. *Phys. Rev. B* **86**, 045314 (2012).
[8] Onsager, L. Interpretation of the de Haas-van Alphen Effect. *Philos. Mag.* **43**, 1006 (1952).
[9] Lifshitz, E. M. & Pitaevskii, L. P. Statistical Physics. Pergamon Press, Oxford (1986).
[10] Kaganov, M. I. & Slutskin, A. A. Coherent magnetic breakdown. *Phys. Rep.* **98**, 189 (1983).
[11] Schoenberg, D. Magnetic Oscillations in Metals. Cambridge University Press, London (1984).
[12] Taskin, A. A. & Ando, Y. Berry phase of nonideal Dirac fermions in topological insulators. *Phys. Rev. B* **84**, 035301 (2011).
[13] Mikitik, G. P. & Sharlai, Yu. V. Berry phase and the phase of the Shubnikov-de Haas oscillations in three-dimensional topological insulators. *Phys. Rev. B* **85**, 033301 (2012).
[14] Murakawa, H. *et al.* Detection of Berry's Phase in a Bulk Rashba Semiconductor. *Science* **342**, 1490 (2013).
[15] Zhao, Y. *et al.* Anisotropic Fermi Surface and Quantum Limit Transport in High Mobility Three-Dimensional Dirac Semimetal Cd_3As_2. *Phys. Rev. X* **5**, 031037 (2015).
[16] Berry, M. V. Quantal Phase Factors Accompanying Adiabatic Changes. *Proc. R. Soc. Lond. A* **392**, 45 (1984).
[17] Mikitik, G. P. & Sharlai, Yu. V. Manifestation of Berry's Phase in Metal Physics. *Phys. Rev. Lett.* **82**, 2147 (1999).
[18] Zak, J. Berrys phase for energy bands in solids. *Phys. Rev. Lett.* **62**, 2747 (1989).
[19] Xiao, D., Chang, M. C. & Niu, Q. Berry phase effects on electronic properties. *Rev. Mod. Phys.* **82**, 1957 (2010).
[20] Novoselov, K. S. *et al.* Two-dimensional gas of massless Dirac fermions in graphene. *Nature* **438**, 197 (2005).
[21] Zhang, Y., Tan, Y.-W., Stormer, H. L. & Kim, P. Experimental observation of the quantum Hall effect and Berry's phase in graphene. *Nature* **438**, 201 (2005).
[22] Luk'yanchuk, I. A. & Kopelevich, Y. Dirac and normal fermions in graphite and graphene: implications of the quantum Hall effect. *Phys. Rev. Lett.* **97**, 256801 (2006).
[23] Castro Neto, A. H., Guinea, F., Peres, N. M. R., Novoselov, K. S., Geim, A. K. The electronic properties of graphene. *Rev. Mod. Phys.* **81**, 109 (2009).
[24] Huang, X. *et al.* Observation of the Chiral-Anomaly-Induced Negative Magnetoresistance in 3D Weyl Semimetal TaAs. *Phys. Rev. X* **5**, 031023 (2015).
[25] Hu, J. *et al.* π Berry phase and Zeeman splitting of Weyl semimetal TaP. *Sci. Rep.* **6**, 18674 (2016).
[26] Sergelius, P. *et al.* Berry phase and band structure analysis of the Weyl semimetal NbP. *Sci. Rep.* **6**, 33859 (2016).
[27] He, L. P. *et al.* Quantum Transport Evidence for the Three-Dimensional Dirac Semimetal Phase in Cd_3As_2. *Phys. Rev. Lett.* **113**, 246402 (2014).
[28] Zheng, G. *et al.* Transport evidence for the three-dimensional Dirac semimetal phase in $ZrTe_5$. *Phys. Rev. B* **93**, 115414 (2016).
[29] Wright, A. R. & McKenzie, R. H. Quantum oscillations and Berry's phase in topological insulator surface states with broken particle-hole symmetry. *Phys. Rev. B* **87**, 085411 (2013).
[30] Yan, Y. *et al.* High-Mobility Bi_2Se_3 Nanoplates Manifesting Quantum Oscillations of Surface States in the Sidewalls. *Sci Rep.* **4**, 3817 (2014).
[31] Liu, H., Liu, S., Yi, Y., He, H. & Wang, J. Shubnikov-de Haas oscillations in n and p type Bi_2Se_3 flakes. *2D Materials* **2**, 045002 (2015).

[32] Devidas, T. R. *et al.* Role of Se vacancies on Shubnikov-de Haas oscillations in Bi_2Se_3: A combined magneto-resistance and positron annihilation study. *Europhysics Lett.* **108**, 67008 (2014).
[33] Hyde, G. R., Beale, H. A., Spain, I. L. & Woollam, J. A. Electronic properties of Bi_2Se_3 crystals. *J. Phys. Chem. Solids* **35**, 1719 (1974).
[34] Barua, S., Rajeev, K. P. & Gupta, A. K. Evidence for topological surface states in metallic single crystals of Bi_2Te_3. *J. Phys.: Condens. Matter* **27**, 015601 (2015).
[35] Tang, H., Liang, D., Qiu, R. L. J. & Gao, X. P. A. Two-Dimensional Transport Induced Linear Magneto-Resistance in Topological Insulator Bi_2Se_3 Nanoribbons. *ACS Nano* **5**, 7510 (2011).
[36] Hajdu, J. & Landwehr, G. Quantum Transport in Semiconductors in High Magnetic Fields. Topics in Applied Physics Vol. **57**, Springer-Verlag, Berlin (1985).
[37] Ibach, H. & Lüth, H. Solid-State Physics. Springer-Verlag, Berlin (2009).
